# Fraction from *Calliandra portoricensis* reduces 7, 12 dimethylbenz(a)anthracene-induced mammary tumors in Wistar rats

**DOI:** 10.22038/AJP.2021.18641

**Published:** 2022

**Authors:** Samson O. Kosemani, Aminat A. Bakare, Oluwatosin A. Adaramoye

**Affiliations:** 1 *Department of Biochemistry, Faculty of Basic Medical Sciences, College of Medicine, University of Ibadan, Ibadan, Nigeria*; 2 *Department of Biochemistry, BOWEN University, Iwo, Nigeria*

**Keywords:** Apoptosis, Calliandra portoricensis Dimethylbenz(a)anthracene Mammary gland

## Abstract

**Objective:**

*Calliandra portoricensis *(CP) is used in Nigeria for the treatment of breast diseases. We investigated the effects of fraction from CP on 7, 12-dimethylbenz(a)anthracene (DMBA)-induced mammary gland tumors.

**Materials and Methods:**

Female Wistar rats (40) were allotted into five equal groups. Group 1 served as control, group 2 received DMBA (50 mg/kg), groups 3 and 4 received DMBA and were treated with CP at doses of 50 and 100 mg/kg respectively, and the group 5 received DMBA and vincristine (0.5 mg/kg). DMBA was injected intraperitoneally once while vincristine and CP were given twice and thrice per week, respectively.

**Results:**

Administration ofDMBA caused a significant decrease in body weight gain by 52%. In addition, DMBA significantly increased organo-somatic weight of mammary gland by 4.0 folds. Also, DMBA significantly increased inflammatory and oxidative stress markers serum interleukin-1β (IL-1β), lipid peroxidation (LPO) and myeloperoxidase (MPO) by 27, 18 and 435%, respectively. Similarly, mammary NO (nitric oxide) and LPO were increased by 468 and 21%, respectively. In contrast, DMBA decreased the levels of apoptotic markers BAX, caspases 3 and 9 by 20, 15 and 18%, and mammary superoxide dismutase (SOD), catalase (CAT) and glutathione-s-peroxidase (GPx) by 45, 51 and 68%, respectively. Histology revealed gland with malignant epithelial cells and high nucleo-cytoplasm in DMBA-administered rats. Treatment with CP 100 mg/kg decreased LPO, MPO, IL-1β and NO by 28, 35, 78 and 85%, respectively, and ameliorated DMBA-induced cyto-architectural anomalies.

**Conclusion:**

Fraction of CP protects mammary gland from DMBA insults via antioxidative and anti-inflammatory mechanisms.

## Introduction

Mammary gland neoplasm is a complex disease with unresolved scientific explanation for its origin. Several risks factors have been linked to the possible causes of this disease such as reproductive and post-menopausal age, exposure to carcinogens, and western-lifestyle (Azubike et al., 2018 ▶). Globally, mammary gland neoplasm is the foremost basis of cancer-based mortality in women without discrimination to ethnicity and race (Yedjou et al., 2019 ▶). In 20 years and more, occurrence and deaths as a result of this disease were comparatively stable in developed countries. Conversely, the case is opposite in Africa, Asia, and central and South America (Youlden et al., 2012 ▶). In Africa, occurrence of mammary gland neoplasm ranged from twenty-seven per a hundred thousand in central Africa to thirty-nine per hundred thousand women in southern Africa in 2012 (Ferlay et al., 2012 ▶). On the other end, the rate of mortality from the disease in the Sub-Saharan Africa is very high due to late presentation of cases, poor health facilities, lack of funding and poor public health awareness (Pace and Shulman, 2016 ▶).

 Treatment options for the management of this ailment include surgery, radiotherapy, chemotherapy, hormonal therapy and immunotherapy (Akram et al., 2017 ▶). These treatment regimens have deleterious effects and patients have been observed to develop complications from such treatments (Senapati et al., 2018 ▶).

 Novel strategies in the treatment of breast cancer are developing; these include immune metabolic engineering, epigenetic therapies such as the use of DNA methyltransferase inhibitors which can upregulate the expression of tumor suppressor genes that were abnormally silenced in cancer cells (Jones et al., 2016 ▶; Yau et al., 2018 ▶) and precision medicine.

 Treatment using chemotherapy/immunotherapy in combination with engineering of the tumor micro-environment has been reported to induce cell death (O’Brien and Finlay, 2019 ▶). In addition, the development of high-performance computational analysis has helped in the area of ‘omics’ which has proven to be a useful tool in better prognosis and identification of therapeutic target in the treatment of breast cancer (Browne et al., 2018 ▶, Ward et al., 2019 ▶, Varešlija et al., 2019 ▶). Therefore, drug discovery and development from natural products, which are known to be safer, pharmacologically active, less toxic and cost effective, should be encouraged as a novel strategy and, is the main focus of many cancer researchers from developing countries. Africa is blessed with vast majority of natural products used for medicinal purposes. Calliandra portoricensis (CP) is a perennial shrub of about 6m tall with ever green small pinnate leaves, which is grown in Asia, South America, Nigeria and other West African nations. Itbelongs to the family Mimosaceae and is markedly ornamental, with white semicircular flower heads and fern-like foliage (Nvau et al., 2020 ▶). The leaves and root barks are widely used in traditional herbal homes. It is prescribed by Nigerian traditional healers as a worm emitter, diuretic and aborticide. Several studies have confirmed the anticonvulsant (El Ghani, 2006 ▶), analgesic (Falode et al., 2018 ▶), cytotoxic, anti-sickling (Amujoyegbe et al., 2012 ▶), antimicrobial and antiproliferative properties (Adaramoye et al., 2015 ▶) of CP. Previous reports from our studies demonstrated that phytochemicals from methanol and chloroform extracts of CP act against prostate hyperplasia, mammary gland carcinogenesis and reproductive toxicity (Adaramoye et al., 2015 ▶; Adefisan et al., 2019 ▶; Adefisan et al., 2020 ▶). 

The present work was designed to examine the protective properties of C. portoricensis on DMBA-induced mammary gland tumors in female rats with insight into its antioxidative, apoptotic and anti-inflammatory actions.

## Materials and Methods

**Collection and extraction of CP**

Root of CP was collected in October 2019 from a community in Osogbo, Osun State, Nigeria and authentication was done at the Herbarium, Department of Botany, University of Ibadan with voucher specimen number UI-012678. It was air-dried, and pulverized and the powdered form was defatted using n-hexane and later subjected to cold extraction using absolute methanol. The methanol extract was subjected to fractionation to obtain the chloroform fraction of CP. The chloroform fraction at 40°C was concentrated in vacuum using rotary evaporator, to dryness. The extraction yielded 5.9% of chloroform fraction of CP. 

**Experimental animals**

Five-week old female Wistar rats that weighed 30-40 g, were obtained from the Animal Facility of the Veterinary Medicine Department, University of Ibadan, Nigeria. The animals were maintained at 25±3°C, and 60±10% relative humidity with a 12-hr light/dark cycle and kept in plastic cages. They were given feed and water *ad libitum*. The feed or laboratory chow was purchased from Ladokun Feeds Industry, Ibadan, Nigeria. All experimental techniques were performed according to the NIH Guidelines for the Care and Use of Laboratory Animals. Experimental protocol, rats handling and treatment were approved by the University of Ibadan Animals’ Ethics Committee (UI-ACUREC/App/2015/061).

**Chemicals**

Dimethyl benzo(a)anthracene (DMBA) was gotten from Sigma, St. Louis, MO, USA and was kept in the dark at 4°C**.** Reduced glutathione, hydrogen peroxide, 5, 5’-dithios-bis-2-nitrobenzoic acid (DTNB), *O*-dianisidine, and epinephrine were procured from Sigma Chemical Co., Saint Louis, MO USA, while trichloroacetic acid (TCA) and thiobarbituric acid (TBA) were obtained from British Drug House (BDH) Chemical Ltd., Poole, UK. Vincristine was procured from a resident pharmaceutical outlet in Ibadan, Nigeria. The remaining chemicals were of analytical grade.

**Study design**

Forty female Wistar rats (Five weeks old) were allotted into 5 groups of eight animals each: Group 1 animals were given normal saline (control), group 2 received DMBA (50 mg/kg), DMBA was initially given to groups 3 and 4, and then they were treated with *Calliandra portoricensis* (50 and 100 mg/kg respectively), while group 5 received DMBA (50 mg/kg) and vincristine (0.5 mg/kg). A sole dosage of DMBA was injected in the intraperitoneal region at 7 weeks of age. *C.portoricensis* was administered by gavage thrice a week, whereas vincristine (VIN) was administered intraperitoneally twice per week for a period of 12 weeks. The dosage of VIN is based on the studies of Adefisan et al. (2019 and 2020). The animals were sacrificed by cervical dislocation. Blood was collected through ocular puncture and mammary tissues were excised for biochemical analyses.

**Preparation of tissues.**

After the last dose, the animals were fasted overnight and then sacrificed. Mammary tissues were excised, rinsed in 1.15% KCl solution (ice-cold), dried and weighed. A portion of the tissue from each group was fixed in formalin (10%) for histological examination. Other portion of the mammary tissue was homogenized in 10 volumes of 50 mM phosphate buffer (pH 7.4) using a Teflon homogenizer. The homogenates were spun using ice-cold ultracentrifuge equipment for 15 min at 10,000 g to obtain post-mitochondrial fraction (PMF) which was used for biochemical analyses. 

**Biochemical assays**

**Protein determination**

The protein levels were assessed by the method of Lowry et al. (1951), using BSA (bovine serum albumin) as a standard. Briefly, Lowry reagent (0.7 ml) was added to the diluted sample/ BSA standard (0.5 ml), then incubated (at room temperature for 20 min). Afterwards, Folin’s reagent (0.1 ml) (diluted) was added, mixed via prompt vortexing and incubated for another 30 min. Next, the absorbance (at 750 nm) was taken by means of a SpectraMaxTM M3 Plate Reader (Molecular Devices, San Jose, CA). Through BSA calibration curve by simple extrapolation, the protein values were determined.

**Assay of superoxide dismutase (SOD)**

The functional SOD activity in the mammary tissue was determined by the method of McCord and Fridovich (1969) ▶ based on the inhibition of autoxidation of epinephrine (pH 10.2) at 30°C. The assay mixture contained carbonate buffer (pH 10.2), 1.5 ml of 0.05 M and the sample (20 μl). The mixture was allowed to equilibrate in the spectrophotometer. Newly-prepared 0.3 mM adrenaline (0.3 ml) was added and mixed by inversion. The increase in absorbance (480 nm) was observed spectrophotometrically at intervals (30 sec each for 150 sec) and SOD specific activity is expressed as µmol/mg protein.

**Assay of catalase (CAT)**

The activity of catalase was determined by method described by Aebi (1974). About 2.4 ml of phosphate buffer (50 mM, pH 7.0), 50 μl of sample and 1.0 ml of 19 mM of hydrogen peroxide (H_2_O_2_) were allowed to run (for 3 min). Subsequently, by addition of 2 ml of dichromate/acetic acid solution, the reaction was terminated and the process was followed by heating (for 10 min in a boiling water bath). The mixture was cooled to 25^o^C, and the decrease in absorbance (at 570 nm) was measured in a spectrophotometer. The catalase activity is expressed as µmol/mg protein.

**Assay of glutathione peroxidase (GPx)**

GPx activity was assessed by the method of Rotruck et al. (1973) ▶. The reacting mixture contained 0.5 ml of sodium phosphate buffer (solution), 0.1 ml of sodium azide (10.0 mM), 0.2 ml of reduced glutathione (GSH) (4.0 mM), 0.1 ml of H_2_O_2 _(2.5 mM) and 0.5 ml of the sample. This was later made-up to 2.0 ml with distilled water and incubated in a water bath at 37°C (for 3 min). Subsequently, 0.5 ml of TCA (10%) was added to discontinue the reaction and the mixture was spun. The resulting supernatant was used to determine reduced glutathione (GSH) content by adding 1.0 ml of disodium hydrogen phosphate (Na_2_PO_4_) (0.3 M) and 0.25 ml of 5, 5"-dithiobis-2-nitrobenzoic acid (DTNB). The optical density was estimated and glutathione peroxidase (GPx) activity was determined. 

**Reduced glutathione (GSH)**

Level of GSH was evaluated by the method described by Moron and colleagues (1979) ▶. Briefly, to an aliquot of mammary tissue post-mitochondria fraction (PMF), sulfosalicylic acid (4%) was added to deproteinize and the resultant mixture was centrifuged at a speed of 3,000 g for 15 min. The supernatant (500 μl) obtained was added to 50 μl of DTNB solution. Ultimately, GSH level was proportional to optical density at 412 nm. 

**Myeloperoxidase (MPO)**

Myeloperoxidase (MPO) activity was evaluated by the adapted method of Trush and his colleagues (1994). Myeloperoxidase activity was assessed spectrophotometrically by reacting *O*-dianisidine with H_2_O_2_. MPO catalyzes the oxidation of *O*-dianisidine in the presence of H_2_O_2 _(acting as an oxidising agent) to produce a brown-coloured product, and absorbs maximally at 470 nm wavelength.

**Nitric oxide**

Serum concentration of NO_3_^-^ and NO_2_^-^ were assessed as an index of nitric oxide (NO) production by the technique of Palmer et al. (1987) ▶. The amount of nitrite (NO_2_^-^) in the mammary tissue post-mitochondrial fraction was assessed following Griess’ reaction. The reactions contained 0.5 ml of sample and 0.5 ml of Griess’ reagent, which was incubated at 25^o^C for 20 min. The optical density was evaluated at 550 nm and the concentration of NO_2_^- ^(nitrite) was assessed by extrapolation from a standard curve made for sodium nitrite (of known concentration).

**Lipid peroxidation (LPO) assay**

Formation of peroxide in the mammary tissue was assessed by the method described by Buege and Aust (1978) ▶. In brief, 0.4 ml of sample was reacted with 1.6 ml of Tris-KCl (solution) and 0.5 ml of TCA (30%). Subsequently, 0.5 ml of TBA (0.75%) was added to the mixture and placed in a hot water bath at 80^o^C for a period of 45 min. The mixture was allowed to cool and centrifuged at 3000 g for 15 min. The optical density of the resulting supernatant was measured using a spectrophotometer (at 532 nm) against the blank.

**Assessment of apoptotic and inflammatory indices**

The levels of caspases 3 and 9, Bax and interleukin (IL)-1β were assessed using ELISA method following manufacturers’ instructions in the diagnostic kits.

**Statistical analysis**

Data are expressed as the MeanSD of eight animals per group (n=8). The values were evaluated using one-way ANOVA followed by the *post-hoc* Duncan’s multiple range test for analysis of biochemical data using SPSS (23.0). Values were considered statistically significant at p<0.05.

## Results

**Assessment of the effects CP on body weight, inflammation and oxidative stress in DMBA-administered rats**

**Figure 1:**
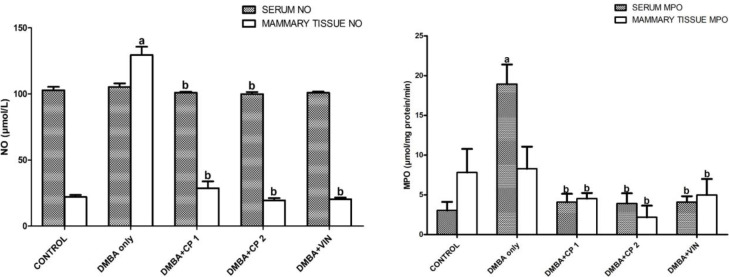
Effect of *Calliandra portoricensis* on the activities of serum and mammary nitric oxide (NO) and myeloperoxidase (MPO) in DMBA-induced mammary gland neoplasm in female rats.Values are expressed as mean±SD of 8 animals. DMBA=7, 12-dimethylbenz-[a] anthracene, CP=Calliandra portoricensis VIN=Vincristine, CP 1=Calliandra portoricensis at a dose of 50 mg/kg, CP 2=Calliandra portoricensis at a dose of 100 mg/kg. ^a^significantly different from CONTROL (p<0.05). ^b^significantly different from DMBA only (p<0.05)

**Figure 2:**
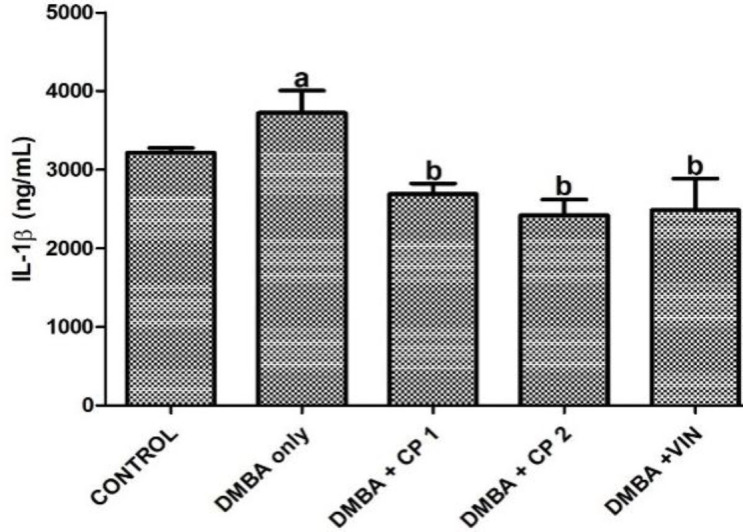
Effect of *Calliandra portoricensis* on the expression of serum interleukin-1β (IL-1 β) in DMBA-induced mammary gland neoplasm in female rats. Values are expressed as mean±SD of 8 animalsDMBA=7, 12-dimethylbenz-[a] anthracene, CP=Calliandra portoricensis VIN=Vincristine, CP 1=Calliandra portoricensis at a dose of 50 mg/kg, CP 2=Calliandra portoricensis at a dose of 100mg/kg. ^a^significantly different from CONTROL (p<0.05). ^b^significantly different from DMBA only (p<0.05).

**Figure 3:**
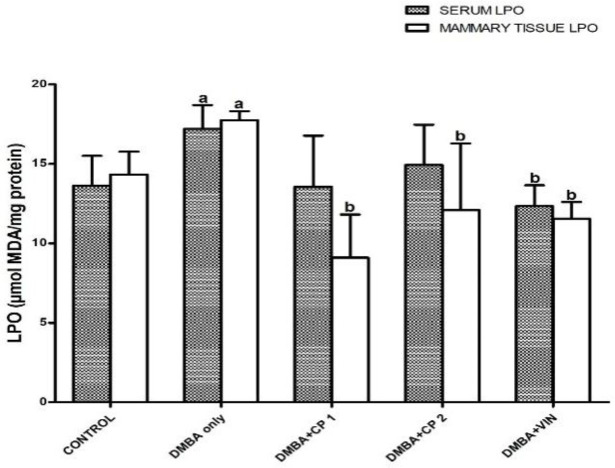
Effect of *Calliandra portoricensis* on the activities of mammary lipid peroxidation (LPO) in DMBA-induced mammary gland neoplasm infemale rats.Values are expressed as mean±SD of 8 animals DMBA=7, 12-dimethylbenz-[a]anthracene,CP=*Calliandra portoricensis* VIN=Vincristine, CP 1=*Calliandra portoricensis* at a dose of 50 mg/kg, CP 2=*Calliandra portoricensis* at a dose of 100 mg/kg. ^a^significantly different from CONTROL (p<0.05).^ b^significantly different from DMBA only (p<0.05).

**Figure 4:**
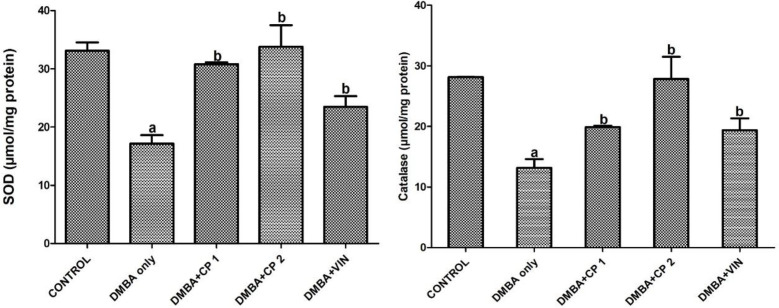
Effect of *Calliandra portoricensis* on the activities of mammary superoxide dismutase (SOD) and catalase (CAT) in DMBA-induced mammary gland neoplasm in female rats. Values are expressed as mean±SD of 8 animals. DMBA=7, 12-dimethylbenz-[a] anthracene, CP=*Calliandra portoricensis,* VIN=Vincristine, CP 1=*Calliandra portoricensis* at a dose of 50 mg/kg, CP 2=*Calliandra portoricensis* at a dose of 100 mg/kg. ^a^significantly different from CONTROL (p<0.05). ^b^significantly different from DMBA only (p<0.05).

**Figure 5:**
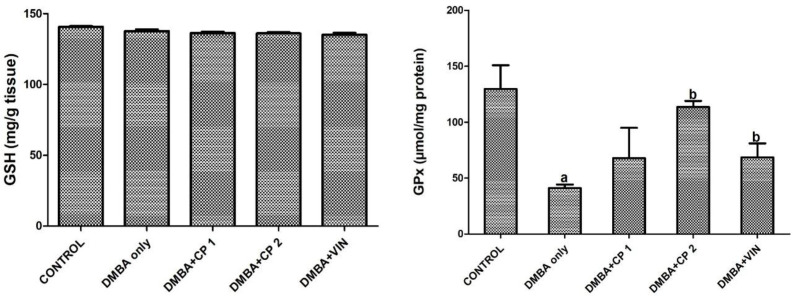
Effect of *Calliandra portoricensis* on the activities of mammary reduced glutathione (GSH) and glutathione peroxidase (GPx) in DMBA-induced mammary gland neoplasm in female rats. Values are expressed as mean±SD of 8 animals. DMBA=7, 12-dimethylbenz-[a] anthracene, CP=*Calliandra portoricensis* VIN=Vincristine, CP 1=*Calliandra portoricensis* at a dose of 50 mg/kg, CP 2=*Calliandra portoricensis* at a dose of 100 mg/kg. ^a^significantly different from CONTROL (p<0.05). ^b^significantly different from DMBA only (p<0.05)

Table 1 shows that body weight gain of the rats given DMBA decreased significantly (p<0.05) by 52%, while the organo-somatic weight of mammary gland increased by 291% relative to the control. Nevertheless, treatment with CP (50 mg/kg) significantly (p<0.05) increased body weight gain and decreased the mammary organo-somatic weight when compared to the DMBA-group. In Figure 1, it is shown that the activity of serum MPO and level of mammary NO increased notably by 2.6 and 4.3 folds, respectively relative to control. Similarly, the level of IL-1β increased significantly (p<0.05) by 27% relative to the control (Figure 2). However, CP (50 and 100 mg/kg) administration to DMBA-administered animals resulted to a significant decrease in serum MPO, IL-1β and mammary NO when compared to the DMBA-group. Furthermore, administration of DMBA caused a significant increase in the serum and mammary lipid peroxidation (LPO) levels by 18% and 21%, respectively relative to the control (Figure 3). However, co-administration of DMBA and CP (50 and 100 mg/kg) significantly reduced the DMBA-induced increase in mammary LPO by 49 and 32%, respectively when compared to the DMBA-group. Figures 4 and 5 show that there were substantial decreases (p<0.05) in mammary SOD, CAT and GPx activities by 45, 51and 68%, respectively relative to the control. Co-administration of DMBA and CP (100 mg/kg) increased the activities of these mammary antioxidative indices relative to the DMBA-group. In addition, mammary GSH levels across treated groups remained statistically similar (p>0.05) when compared to the control.

**Table 1:** Effects of *Calliandra portoricensis* on body weight and relative body weight of DMBA-induced mammary cancer in female rats

Treatments	Initial body Wt (g)	Final body Wt (g)	Body Wt gain (g)	Weight of Mammary Tissue (g)	Organo-somatic Wt (as % body wt)
CONTROL	41.75±0.96	185.5±27.74	143.75±26.81	8.17±1.45	4.42±0.64
DMBA only	59.5±5.07	127.25±14.73	67.75±17.13^a^	21.96±0.45	17.26±1.33^a^
DMBA+CP1	47.25±2.75	162±14.49	114.75±12.31^b^	11.56±7.60	6.91±4.21^b^
DMBA+CP2	48.75±5.56	128.25±37.30	79.5±32.25	9.82±0.68^b^	7.66±0.83^b^
DMBA+VIN	62±3.83	148.25±21.09	86.25±18.48	14.99±0.20	10.11±1.19^b^

Values are expressed as mean±SD of 8 animals.^a^significantly different from CONTROL (p<0.05).^b^significantly different from DMBA only (p<0.05). Wt=Weight, Diff=Difference. DMBA=7, 12-dimethylbenz-[a] anthracene, CP=*Calliandra *portoricensis, VIN=Vincristine. CP 1=*Calliandra portoricensis* at a dose of 50 mg/kg, CP 2=*Calliandra portoricensis* at a dose of 100 mg/kg

**Effect of CP on apoptosis and histology of mammary tissue of DMBA administered rats**

**Figure 6:**
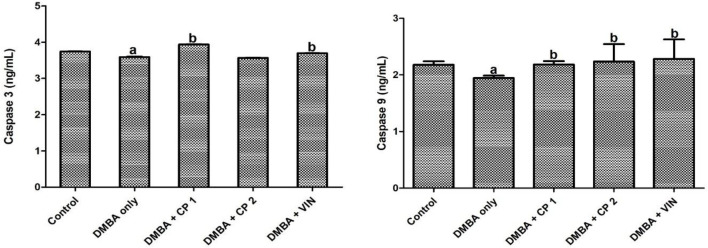
Effect of *Calliandra portoricensis* on the expression of serum caspase 3 (Cas3) and caspase 9 (Cas9) in DMBA-induced mammary gland neoplasm in female rats. Values are expressed as mean±SD of 8 animals. DMBA=7, 12-dimethylbenz-[a] anthracene, CP=*Calliandra portoricensis*, VIN=Vincristine, CP 1=*Calliandra portoricensis* at a dose of 50 mg/kg, CP 2=*Calliandra portoricensis* at a dose of 100 mg/kg. ^a^significantly different from CONTROL (p<0.05). ^b^significantly different from DMBA only (p<0.05).

**Figure 7:**
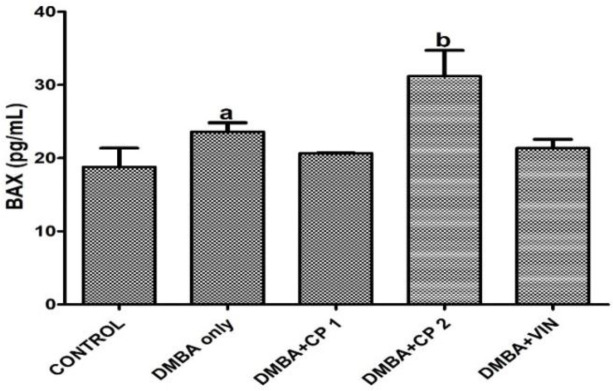
Effect of *Calliandra portoricensis* on the expression of serum Bcl-2-associated X protein (BAX) in DMBA-induced mammary gland neoplasm in female rats. Values are expressed as mean±SD of 8 animals. DMBA=7, 12-dimethylbenz-[a] anthracene, CP=*Calliandra portoricensis*, VIN=Vincristine, CP 1=*Calliandra portoricensis* at a dose of 50 mg/kg, CP 2=*Calliandra portoricensis* at a dose of 100 mg/kg. ^a^significantly different from CONTROL (p<0.05). ^b^significantly different from DMBA only (p<0.05).

In Figure 6, it is shown that caspase 3 (Cas 3) and caspase 9 (Cas 9) levels were significantly (p<0.05) reduced in DMBA-administered rats, whereas co-administration with CP increased the levels of these caspases. As shown in Figure 7, administration of DMBA did not statistically alter the level of Bax in DMBA group in relation to the control. However, co-administration of DMBA and CP (100 mg/kg) significantly (p<0.05) increased Bax level relative to the DMBA group.

**Figure 8:**
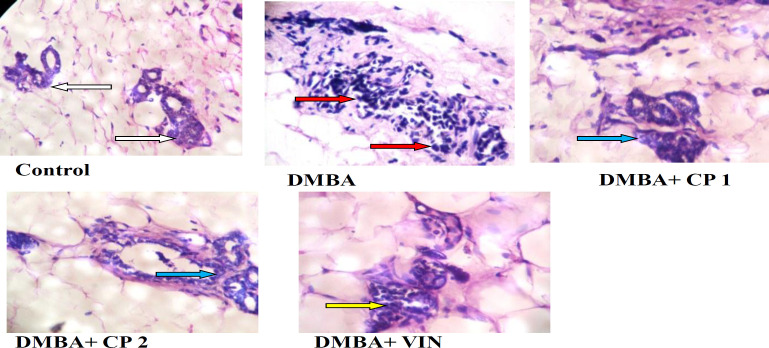
Representative photomicrographs showing the effect of *Calliandra portoricensis* (CP) on mammary tissues of rats treated with 7, 12-dimethylbenz-[a] anthracene (DMBA). VIN=Vincristine, CP 1=*Calliandra portoricensis *at a dose of 50 mg/kg, CP 2=*Calliandra portoricensis *at a dose of 100 mg/kg.White arrow: Normal epithelial cells. Red arrow: presence of benign adenomas. Blue arrow: mammary glands with mild hyperplasia. Yellow arrow: mammary glands with moderate hyperplasia

Figure 8 depicts that DMBA-administered rats had moderately increased periductal fibrous tissues and benign fibro-adenoma compared to the control which displays the existence of normal stroma and mammary adipose tissues. However, co-administration of DMBA and CP attenuated the periductal fibrous tissues and completely removed the benign fibro-adenomas. Similar effects were observed in mammary slides of rats given DMBA and VIN.

## Discussion

Plant-based medicines have proven to be great relief in the management of diseases and infection in the healthcare system (Pop et al., 2018 ▶). This is due to the fact that they are almost readily accessible, easily processed, safer/ non-toxic, effective and economical (Sen and Samanta, 2014 ▶). *Calliandra portoricensis* (CP) is one medicinal plant used profoundly in the South-western part of Nigeria. Previous studies have shown CP to have anti-tumorigenic effects in cells of prostate cancer (Oyebode et al., 2018 ▶; Adefisan et al., 2019 ▶). In the present study, by means of histological and biochemical assessments, we evidently established that CP possesses effective anti-neoplastic effects to counter DMBA-induced mammary gland neoplasm in experimental animal model. Weight loss is one of the most common indicators of disease progression and it is most commonly seen in cancer and diabetes. The weight loss could be attributed to physiological anomalies at primary tumor sites and gastrointestinal syndromes (Sanchez-Lara et al., 2013 ▶). In the present study, DMBA administration produced weight loss in the animals. This observation has been reported in a study where DMBA was used as a carcinogen in rodents (Rajendran et al., 2019 ▶). However, co-administration with CP was able to restore the weight loss, an indication that CP enhances weight gain. The growth and survival of cancer cells have been linked to oxidative stress at all stages leading to the development of full-blown neoplasm (Podgorski et al., 2020 ▶).

The link between DMBA toxicity and oxidative has been established. However, proper functioning of cells including cancer cells requires a definite amount of reactive oxygen species (ROS) to perform normal physiological functions, whereas over-production of ROS can deplete endogenous antioxidant defense mechanism in normal cells which will alter cellular homeostasis (Almokhtar et al., 2019 ▶). Therefore, moderate concentration of ROS is physiologically required in different cells of the body. On the contrary, oxidative stress caused by ROS induces a redox unevenness in many cancerous cells as and thus, leads to oncogenic stimulation. As a result, oxidative stress causes permanent damage to genetic materials which leads to cell arrest or induction of transcription, induction of signal transduction pathways, replication errors, and genomic instability, all of which are associated with carcinogenesis (Marnett, 2000 ▶; Valko et al., 2006 ▶). In this study, our results show that exposure of experimental rats to DMBA significantly induced oxidative stress as revealed by the elevation of malonaldehyde levels. This observation is in agreement with data published by Mani et al. (2018) ▶ who reported increased malondialdehyde generation and consequent oxidative stress on exposure of rats to DMBA. Antioxidants are predominantly the body’s tools to detoxify ROS (Nugent, 2019 ▶). Antioxidant systems in the body operate in different levels according to the line of defense. Superoxide dismutase (SOD) and catalase (CAT) are members of the first line or primary defense system (Mao et al., 2019 ▶). They are so named because they rapidly detoxify primary ROS molecules that are capable of forming free radicals or they have the potential of mitigating the formation of other radicals (Ighodaro and Akinloye, 2018 ▶). In this study, the antioxidative role of CP led to a substantial reduction in LPO level, and an increase in SOD and CAT activities in the rats exposed to DMBA. These findings correlated with the findings of the work of Mani et al. (2018) ▶ who reported a significant decrease in the level of mammary LPO in DMBA-induced neoplasm in the mammary gland of the rats. The decrease in SOD and CAT activities in DMBA-administered rats could be attributed to high production of ROS which have been implicated in the initiation of carcinogenesis and the progression of mammary gland neoplasm caused by this chemical (Aggarwal et al., 2019 ▶). Glutathione peroxidase, GPx breaks down lipid peroxides to their respective alcohol and hydrogen peroxide to water principally in the mitochondria, and therefore protects cells from oxidative stress (Zhang et al., 2019 ▶). In our study, a decrease in the activity of GPx in DMBA group was observed. This is in agreement with the study of Faheem and Elnbtete, (2020) ▶ who remarked that DMBA could cause a decline in the mammary GPx activities in experimental animals during the development of mammary neoplasm. Interestingly, treatment with CP restored the activities of GPx which further confirms antioxidant activity of this medicinal plant. 

Inflammation plays a key part in tumor advancement and consequently high level of nitric oxide and other inflammatory agents are found in mammary tissues undergoing inflammation (Munn, 2016 ▶). This in part may account for formation of NO by nitric oxide synthase (NOS) in immunological cells during the adenoma of pre-neoplastic transformation in some tissues (Ambs et al., 1998 ▶). Similarly, during the process of inflammation, vascular permeability is amplified by stimulation of inflammatory mediators (Phillipson and Kubes, 2011 ▶), and this may lead to invasion of polymorphonuclear neutrophils and immunoglobulins,consequently giving rise to the release of myeloperoxidase (MPO) enzyme (Selders et al., 2017 ▶). In the present study, DMBA administration led to an upsurge in MPO activities and NO levels in the mammary glands. The result is in consonance with the report of Gasparoto et al. (2012) ▶ who notably attributed the increase in MPO activity and NO levels to neutrophils infiltration at the site of mammary gland cancer. Conversely, the ability of CP to mitigate the inflammatory markers justifies its anti-inflammatory properties in a biological system. Interleukin 1β (IL-1β) which is a pro-inflammatory cytokine, is typically implicated in stimulation and development of inflammatory processes and it was assessed in this study. We observed that following exposure of experimental animals to DMBA, the functional level of IL-1β was increased in the rats. In contrast, administration of CP to DMBA rats significantly decreased the level of this protein. This further affirms the anti-inflammatory properties of CP in reducing the incidence of inflammation during DMBA-induced tumors in the tissues. 

Apoptosis as one of the mechanisms by which, natural products can attack and destroy cancer cells, is a physiological process in embryogenesis and tissue homeostasis and it is perfectly coordinated (Millimouno et al., 2014 ▶). In addition, programmed cell death occurs in two major ways: intrinsic (mitochondria-mediated) and extrinsic pathways which could be initiated by many factors. Markers of apoptosis such as caspase 9 and caspase 3 were observed to be significantly reduced in serum of DMBA-administered rats. Importantly, administration of CP at 50 mg/kg increased the caspases level in the rats. This increase in caspases by CP may cause a substantial reduction in mitochondrial membrane potential of the cancer cells and consequently the release of pro-apoptotic protein like cytochrome C from the inter-membrane space thus leading to cell death. The H&E staining of sections of the mammary tumor showed benign fibro-adenoma in the rats given DMBA, in addition to the proliferating malignant ductal epithelial cells. These findings correlate with the study of Bishhayee et al. (2016) ▶ who reported that normal mammary gland differentiation process might be altered by DMBA administration. Administration of CP to DMBA-treated rats, significantly attenuated the benign fibro-adenoma in the mammary tissues. 

In conclusion, fraction of CP reduced the organo-somatic weight of mammary gland, mitigated the oxidative and inflammatory responses and increased the antioxidant enzymes activities of DMBA-administered rats. Additionally, it attenuated the proliferation of mammary ducts and benign fibro-adenoma as revealed by histology. It is recommended that in-depth study be carried out to unravel the underlying molecular and genetic mechanism of the anticancer effects of CP. 

## Conflicts of interest

The authors have declared that there is no conflict of interest.
